# Limited and Short-Lasting Virus Neutralizing Titers Induced by Inactivated SARS-CoV-2 Vaccine 

**DOI:** 10.3201/eid2712.211772

**Published:** 2021-12

**Authors:** Taweewun Hunsawong, Stefan Fernandez, Rome Buathong, Naretrit Khadthasrima, Kamonthip Rungrojchareonkit, Jindarat Lohachanakul, Rungarun Suthangkornkul, Kedsara Tayong, Angkana T. Huang, Chonticha Klungthong, Piyawan Chinnawirotpisan, Yongyuth Poolpanichupatam, Anthony R. Jones, Eric D. Lombardini, Supaporn Wacharapluesadee, Opass Putcharoen

**Affiliations:** Armed Forces Research Institute of Medical Sciences, Bangkok, Thailand (T. Hunsawong, S. Fernandez, K. Rungrojchareonkit, J. Lohachanakul, R. Suthangkornkul, K. Tayong, A.T. Huang, C. Klungthong, P. Chinnawirotpisan, Y. Poolpanichupatam, A.R. Jones, E.D. Lombardini);; Ministry of Public Health, Nonthaburi, Thailand (R. Buathong);; Ministry of Public Health, Samut Sakhon Province, Samut Sakhon, Thailand (N. Khadthasrima);; Thai Red Cross Emerging Infectious Diseases Clinical Center, Bangkok (S. Wacharapluesadee);; Chulalongkorn University Faculty of Medicine, Bangkok (O. Putcharoen)

**Keywords:** coronavirus disease, COVID 19, inactivated SARS**-**CoV**-**2 vaccine, neutralizing antibodies, respiratory infections, SARS-CoV-2, severe acute respiratory syndrome coronavirus 2, Thailand, vaccination, viruses

## Abstract

In vitro determination of severe acute respiratory syndrome coronavirus 2 neutralizing antibodies induced in serum samples from recipients of the CoronaVac vaccine showed a short protection period against the original virus strain and limited protection against variants of concern. These data provide support for vaccine boosters, especially variants of concern circulate.

Circulation of novel severe acute respiratory syndrome coronavirus 2 (SARS-CoV-2) variants capable of evading vaccine-derived protection is challenging the efficacy of coronavirus disease (COVID-19) vaccines (*1*). The inactivated SARS-CoV-2 vaccine CoronaVac (Sinovac Biotech, http://www.sinovac.com), 1 of 2 COVID-19 vaccines licensed in Thailand, has been widely administered to health care workers. Clinical studies show CoronaVac efficacy against symptomatic COVID-19 ranging from 51% (Brazil) to 65.9% (Chile) and 100% against severe illness and illness requiring hospitalization (*2*,*3*). However, data on CoronaVac efficacy against variants of concern are very limited. Our study was approved by the Research Ethics Review Committee, Faculty of Medicine, Chulalongkorn University (Bangkok, Thailand) and recorded in the Thai Clinical Trial Registry **(**TCTR20210325003). Investigators adhered to U.S. Department of Defense AR 70–25 policies for protection of human subjects.

For this study, we enrolled 207 health care workers in Thailand who were fully vaccinated with 2 doses of CoronaVac (0.5 mL/dose, 2–4 wk between doses); all had received their first dose during February 22–March 12, 2021. Median age was 39 (interquartile range 30–51) years of age; 103 (49.6%) were men. Among study participants, 58 (28%) provided blood samples only at baseline (when the first dose was administered), 93 (44.0%) both at baseline and 2–3 weeks after the second dose, and 56 (27.0%) at baseline and at 2–3 weeks and 10–12 weeks after the second dose. Using an in vitro system (Appendix), we evaluated the ability of the serum of CoronaVac recipients to neutralize SARS-CoV-2. We measured circulating serum neutralizing antibodies to the original wild-type strain by using a cPass receptor binding domain antigen-based surrogate virus neutralization test (sVNT) ELISA (GeneScript, https://www.genscript.com) (*4*) and using a microneutralization assay (MNA) (*5*) for SARS-CoV-2 Wild-type strain and Alpha, Beta, and Delta neutralizing antibodies. Seroconversion rates for CoronaVac-vaccinated participants, determined by sVNT ELISA using 30% inhibition as cutoff, were 85.2% (78.2% mean inhibition level) at 2–3 weeks and 35% (25.4% mean inhibition level) at 10–12 weeks. The MNA seropositivity cutoff was set at ≥50%.

At 2–3 weeks after the second dose, 61.1% (91/149) of participants were seropositive against the Wild-type strain, 35.6% (53/149) against Alpha variant, 3.4% (5/149) against Beta, and 8.7% (13/149) against Delta (Figure). Mean neutralizing rate at 2–3 weeks was 49.3% (95% CI 44.9%–53.6%) against Wild-type strain, 40.9% (95% CI 37.8%–43.9%) against Alpha variant, 9.0% (95% CI 6.1%–11.8%) against Beta, and 10.8% (95% CI 7.1%–14.5%) against Delta. At 10–12 weeks after the second dose, the proportion of seropositive participants fell to 50% (28/56) against Wild-type strain and was significantly reduced (p < 0.001) to 17.9% (10/56) against Alpha variant, 1.8% (1/56) against Beta, and 1.8% (1/56) against Delta. Mean neutralizing rates at 10–12 weeks were 48.0% (95% CI 39.9%–56.1%) against Wild-type strain, 21.8% (95% CI 37.8%–43.9%) against Alpha variant, 1.2% (95% CI 3.5%–8.8%) against Beta, and 1.0% (95% CI 2.9%–7.5%) against Delta.

**Figure:**
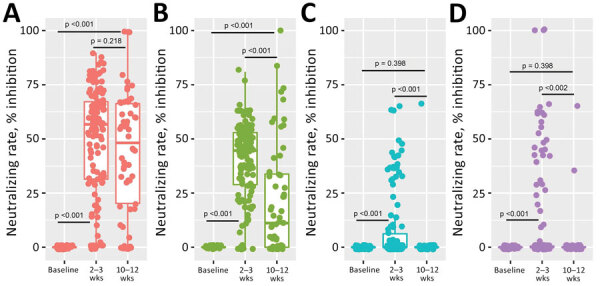
Results of in vitro testing by microneutralization assay of CoronaVac-induced neutralizing A) Wild-type strain and B) Alpha-, C) Beta-, and D) Delta-variant SARS-CoV-2 antibodies (n = 207). Overall vaccine-induced neutralizing antibodies shown at baseline, 2–3 weeks, and 10–12 weeks after second dose. Differences in mean inhibition rate were compared based on blood collection times. p value <0.05 indicates statistical significance.

Comparing sVNT ELISA results between the 2 time points, Wild-type strain antibodies appear to have a half-life of 83.4 days (95% CI 76.6–90.3 days). However, when the MNA was used, neutralizing antibodies waned in a time- and variant-dependent manner. The half-life of neutralizing antibodies was as low as 47.2 days (95% CI 37.5–56.9 days) for Wild-type strain, 38.6 days (95% CI 31.2–45.9 days) for Alpha variant, 6.9 days (95% CI 3.2–10.6 days) for Beta, and 12.3 days (95% CI 6.8–17.8 days) for Delta (Table). These data indicate the possibility that SARS-CoV-2 variants are able to escape humoral induced by wild-type prototype inactivated vaccines, which is consistent with results of other recent studies (*4*,*5*). Our findings support administering vaccine boosters, especially where these variants circulate. 

**Table:** Results of in vitro testing by surrogate virus neutralization test ELISA and microneutralization assay of CoronaVac-induced neutralizing Wild-type strain and Alpha, Beta, and Delta variants of severe acute respiratory syndrome coronavirus 2*

Neutralization test detection method	Slope coefficient (95% CI)	Half-time coefficient, d (95% CI)
Surrogate virus neutralization test ELISA	–0.645 (–0.751 to –0.538)	83.42 (76.55–90.29)
Microneutralization assay		
Wild-type	0.008 (–0.141 to 0.159)	47.17 (37.48–56.86)
Alpha	–0.187 (–0.302 to –0.072)	38.57 (31.16–45.99)
Beta	–0.063 (–0.121 to –0.006)	6.88 (3.20–10.57)
Delta	–0.125 (–0.211 to –0.040)	12.27 (6.78–17.77)

*CoronaVac vaccine by Sinovac Biotech (http://www.sinovac.com).

Appendix Additional information about neutralizing titers in serum of coronavirus vaccine recipients.
