# Supplementary material for: Limited and Short-Lasting Virus Neutralizing Titers Induced by Inactivated SARS-CoV-2 Vaccine
Source: Emerg Infect Dis. 2021 Dec;27(12):3178–80. doi: 10.3201/eid2712.211772 (PMC8632161; doi:10.3201/eid2712.211772)
Supplement: Appendix — Additional information about neutralizing titers in serum of coronavirus vaccine recipients. [file 21-1772-Techapp-s1.pdf]

Article DOI: https://doi.org/10.3201/eid2712.211772

Limited Protection of Inactivated SARS-CoV-2 Vaccine against Wuhan Strain and Variants of Concern

Appendix

Additional Methods

Cell Lines and Viruses

We obtained Vero E6 green monkey kidney epithelial cell line from American Type Culture Collection. Cells were grown in Invitrogen Eagle's minimum essential medium (EMEM; ThermoFisher, https://www.thermofisher.com) supplement with Invitrogen 5% heat-inactivated fetal bovine serum (HIFBS; ThermoFisher) 1% L-glutamine, 1% P&S, 40 µg/mL gentamicin and 0.25 µg/mL fungizone, at 35 ± 2°C, 5% CO_2 incubator. We used 1-day-old cells for measuring median tissue culture infectious dose 50 ($TCID_{50}$) and microneutralization assay.

We obtained SARS-CoV-2 viruses, including the Wuhan linage (Wuhan Hu-1 strain, isolate Hong Kong/VM20001061/2020, NR-52282), Alpha B.1.1.7 variant (hCoV-19/England/204820464/2020, NR-54000, contributed by Bassam Hallis) and Beta B.1.351 variant (hCoV-19/South Africa/KRISP-EC-K005321/ 2020, NR-54008, contributed by Alex Sigal and Tulio de Oliveira) through the National Institute of Allergy and Infectious Diseases Biodefense and Emerging Infections Research Resources Repository (https://www.beiresources.org). We isolated Delta B.1.617.2 variant (hCoV-19/Thailand/CU-A21287-NT/2021) from a clinical specimen collected at King Chulalongkorn Hospital. All isolates were quantitated in Vero E6 cells by $TCID_{50}$ using the Reed-Muench method based on 8 replicates per titration and propagated to generate sufficient titers (100 $TCID_{50}$) for the microneutralization assay.

SARS-CoV-2 Isolation

We completed SARS-CoV-2 isolation in a Biosafety Level (BSL)-3 laboratory at Armed Forces Research Institute of Medical Sciences (https://afrims.amedd.army.mil). We sent the nasopharyngeal specimens with confirmed RT-PCR and genome sequencing for viral isolation.

Virus isolation was done in Vero E6. One day before inoculation, we seeded 1.5 mL of cell suspension at a concentration of 3×10^5 cells/mL onto 5.5 cm^2 tissue culture tubes. We separately inoculated 150 µL of specimen into Vero E6 cells monolayer. After incubation for 1 hr at 37°C in 5% CO_2 incubator, we added 1.5 mL of EMEM supplement with 2% HIFBS, 1% L-glutamine, 1% P&S, 40 µg/mL gentamicin, and 0.25 µg/mL fungizone and continued culturing at 37°C in a 5% CO_2 incubator. We observed the cytopathic effect of virus-infected cells daily. We confirmed variant isolates by PCR and genome sequencing.

Microneutralization Assay

We used a microneutralization assay to determine neutralizing antibodies against SARS-CoV-2 viruses, including the Wuhan strain and variants B.1.1.7 (Alpha), B.1.351 (Beta), and B.1.617.2 (Delta) (*1*). All procedures were performed in a BSL-3 laboratory following a standard neutralization assay using cytopathic effect (CPE)-based colorimetric read-out (*2*). Cell control (CC) and virus control (VC) wells were included. We heat-inactivated serum samples at 56°C for 30 min before making 1:10 dilutions in Invitrogen 2% HIFBS/EMEM media (ThermoFisher). We separately incubated an equal volume of diluted serum with 100 $TCID_{50}$ of SARS-CoV-2 virus at 37°C and inoculated 5% CO_2 incubation for 1 hr. 100 µL of serum-virus mixture into triplicate wells of Vero E6 cells in 96-well plates and incubated at 37°C and 5% CO_2 for 5 days before staining with Sigma 0.02% neutral red (https://www.sigmaaldrich.com) in Invitrogen 1X phosphate-buffered saline (PBS, ThermoFisher). We added lysis solution for 1 hr at room temperature (RT) before measuring optical density (OD) at 540 nm. All obtained OD values against different strains of SARS-CoV-2 have been normalized with the mean OD of baseline sera against a particular strain. We calculated percentages of virus infectivity in VC and samples based on OD of CC, infectivity (%) = (OD of CC–OD of sample) × 100. We calculated percentage of inhibition using inhibition (%) = 100–[(100 × infectivity of sample)/infectivity of VC]. Percentage of inhibition ≥50% is considered a positive cutoff for seroconversion against SARS-COV-2.

Surrogate Neutralizing Antibody ELISA

We performed a cPass surrogate neutralization antibody test (GenScript Biotech, https://www.genscript.com) as described elsewhere (*3*). We coated 96-well plates with hACE2 protein at 100 ng/well in 100 mmol carbonate-bicarbonate coating buffer (pH 9.6). We diluted serum samples at 1:10 and preincubated them with 6 ng of horseradish peroxidase-conjugated

SARS-CoV-2 spike receptor-binding domain for 1 hr at 37°C. We transferred the mixture into hACE2 coated plate and incubated for 1 hr at RT. After removing unbound horseradish peroxidase-conjugated SARS-CoV-2 spike receptor-binding domain and washing with PBS 0.05% tween-20 (PBST) solution 5×, we added TMB substrate and incubated for 30 minutes at RT. An equal volume of TMB stop solution was added to stop the reaction, and we acquired the absorbance reading at 450 nm and 570 nm by spectrophotometer. We calculated percentage of inhibition using the formula, inhibition (%) = (1 – sample OD value/negative control OD value) ×100.

Statistical Analysis

We performed data analysis using R program version 4.0.2 (https://cran.r-project.org/bin/windows/base/old/4.0.2) and SPSS program Version 25 (https://www.ibm.com/support/pages/downloading-ibm-spss-statistics-25). We used a Mann-Whitney U test or Wilcoxon test to compare the mean % inhibition obtained against different SARS-CoV-2 variants and the differences between baseline and convalescent fully vaccinated serum samples. P value <0.05 was considered statistically significant. Pearson correlation coefficient was used to determine the strength of association between surrogate nitrotyrosine ELISA with each microneutralization datum against prototype and variants.

References

1. Planas D, Veyer D, Baidaliuk A, Staropoli I, Guivel-Benhassine F, Rajah MM, et al. Reduced sensitivity of SARS-CoV-2 variant Delta to antibody neutralization. Nature. 2021;596:276–80. PubMed https://doi.org/10.1038/s41586-021-03777-9

2. Manenti A, Maggetti M, Casa E, Martinuzzi D, Torelli A, Trombetta CM, et al. Evaluation of SARS-CoV-2 neutralizing antibodies using a CPE-based colorimetric live virus micro-neutralization assay in human serum samples. J Med Virol. 2020;92:2096–104. PubMed https://doi.org/10.1002/jmv.25986

3. Tan CW, Chia WN, Qin X, Liu P, Chen MI-C, Tiu C, et al. A SARS-CoV-2 surrogate virus neutralization test based on antibody-mediated blockage of ACE2-spike protein-protein interaction. Nat Biotechnol. 2020;38:1073–8. PubMed https://doi.org/10.1038/s41587-020-0631-z
